# Examination of the Behavior of Gravity Quay Wall against Liquefaction under the Effect of Wall Width and Soil Improvement

**DOI:** 10.1155/2014/325759

**Published:** 2014-07-08

**Authors:** Ali Akbar Firoozi, Mohd Raihan Taha, S. M. Mir Moammad Hosseini, Ali Asghar Firoozi

**Affiliations:** ^1^Department of Civil & Structural Engineering, Universiti Kebangsan Malaysia (UKM), 43600 Bangi, Selangor, Malaysia; ^2^Department of Civil & Structural Engineering, Amirkabir University of Technology, Tehran, Iran

## Abstract

Deformation of quay walls is one of the main sources of damage to port facility while liquefaction of backfill and base soil of the wall are the main reasons for failures of quay walls. During earthquakes, the most susceptible materials for liquefaction in seashore regions are loose saturated sand. In this study, effects of enhancing the wall width and the soil improvement on the behavior of gravity quay walls are examined in order to obtain the optimum improved region. The FLAC 2D software was used for analyzing and modeling progressed models of soil and loading under difference conditions. Also, the behavior of liquefiable soil is simulated by the use of “Finn” constitutive model in the analysis models. The “Finn” constitutive model is especially created to determine liquefaction phenomena and excess pore pressure generation.

## 1. Introduction

Liquefaction-induced flow is phenomena associated with soil liquefaction, sometimes resulting in large displacement of order of several meters [1]. It was first found to occur in the Noshiro city, Japan, during the 1983 Nihonkai-chubu earthquake [2]. The technique to measure residual displacement, comparison of aerial photos before and after the earthquake, was then applied to many past earthquakes as well as subsequent earthquakes, and evidences of liquefaction-induced flow were also collected. As a result, liquefaction-induced flow is found not to be extraordinary phenomena but have commonly occurred in many earthquakes.

Consequently, liquefaction-induced flow was found out as an ordinary event that frequently happened in many earthquakes.

The earthquake of Kobe Port in Japan (1995) caused vast destructions in the quay walls and subsequently in the port facilities and the area around the port. Then, extensive research was started concentrating on the dynamic earth pressure on the quay walls, the way liquefaction affected the soil around the walls, and how the excess pore water pressure is developed in the soil around the walls [3, 4].

Modeling the behavior of the structures during the liquefaction of soil is a complex process. In the numerical modeling of the liquefaction, the simulation of excess pore water pressure and the dynamic nonlinear soil behavior are the prerequisites for these model analyses. For this purpose, the relevant pieces of geotechnical software were evaluated and finally the FLAC was selected so as to facilitate the numerical modeling of the gravity quay wall during the process of the liquefaction. The capability of this software for simulation of excess pore water pressure and also having the Finn behavior model which is particularly helpful in modeling the liquefaction behaviors were the reasons behind this selection [5, 6].

Among the relevant factors that directly affect the behavior of gravity quay walls, three were chosen. First, the effect of the increase in the width of the quay wall on the reduction of the wall deformation and the stability control was studied. The relevant diagrams of the deformation of different parts are drawn. In pursuance, the soil around the quay wall was improved and the SPT thereof was increased so as to study the effect of standard penetration number on the behavior of the quay wall. Since the standard penetration number directly influences other soil features such as compression, friction, and soil density (relative density), other soil features in the analytical models were enhanced as the SPT value was increased. Because there was not a comprehensive relationship between change of the soil features and the SPT value, the results of the sand samples which had been tested at the Laboratory of National University of Malaysia have been used. Finally, to study the optimum zone of the soil improvement, different areas in the back side, the front side, and the base of the quay wall were amended and the models thereof were analyzed. The results obtained from the analyses were evaluated and assessed with regard to the optimum and adequate zone for the soil improvement [3, 4, 7].

## 2. Modeling

Analytical models are developed to predict liquefaction characteristics by Martin et al. [8], Liou et al. [9], Finn et al. [10], Katsikas and Wylie [11], Desai [12], and Liyanapathirana and Poulos [13], to name a few. Some of the analytical models adopted effective stress-based approach (e.g., [9–11, 13]), while few of them adopted energy-based approach (e.g., [12]). Experimental studies including cyclic triaxial tests, shaking table tests and centrifuge tests have been conducted for the last four decades (e.g., [14–16]) to validate the theories and better understand the mechanism. Simplified methods to evaluate liquefaction characteristics from SPT and CPT test results are developed by Seed and Idriss [17], Tokimatsu and Seed [18], Robertson and Wride [19], Youd and Idriss [20], and Idriss and Boulanger [21]. Although simplified total stress-based methods are used in general practice for the ease of computation to evaluate the liquefaction potential and associate settlement, these methods are unable to account for the progressive stiffness degradation of soil due to repeated shearing and pore-pressure rise during an earthquake event. As a result, nonlinear site response analysis and dynamic time history analyses are recommended for design of high risk infrastructures such as dams, bridges and nuclear power plants [21]. In this study, three different analytical models by Finn et al. [10], Liou et al. [9], and Katsikas and Wylie [11] are studied to compare the response of saturated sand deposit under earthquake motions.

Rollins and Seed [22] introduced three factors to evaluate the effect of overburden pressure on liquefaction potential. These factors are static shear stress, effective confining pressure and over consolidated ratio (OCR).

*Shear Stress*. Overburden pressure and slope situation may induce anisotropy consolidated condition and cause initial static shear stress in the soil mass. According to related studies, static shear stress may affect soil liquefaction potential directly. Lee and Seed [14] indicated that the liquefaction resistance of soil increases by increase of static shear stress. Increase of initial static shear stress in the soil mass may lead to increase of soil settlement and compression and subsequently, it leads to increase of Cyclic Resistant Ratio (CRR)

(1) CRR=τcyσ′,

where *τ*_*cy*_ = cyclic shear strength and *σ*′ = vertical effective stress.

*Effective Confining Pressure*. Using the results of dynamic tri-axial testing, Peacock and Seed [23] indicated that cyclic shear stress increases by increase of effective confining pressure, but Cyclic Resistance Ratio (CRR) is contrary. Mulilis et al. [24] denoted that Cyclic Resistance Ratio (CRR) may slightly decrease by increase of effective confining pressure. Hynes and Olsen [25] concluded that several factors such as method of deposition, stress history, aging effects and density may affect the influence of confining stress variations on the CRR.

*Overconsolidation Ratio (OCR)*. According to related studies, over-consolidation state is an important effect for soil liquefaction potential. If a soil mass has experienced stresses higher than its current state, it is an over-consolidated soil (OCR > 1)

(2) $$\begin{array}{c} \text{CRR}=\frac{P_{c}}{P_{0}}, \end{array}$$

where *P*_*c*_ = overconsolidation stress and *P*_0_ = current existing stress.

Seed and Idriss [26] showed that the liquefaction resistance increases by increase of the OCR values. Using cyclic torsion shear test, Ishihara and Takatsu [27] showed the relations between OCR, *K*_0_ and cyclic shear strength. As shown in their results, under constant *K*_0_, the cyclic stress ratio increases by increase of the OCR value.

### 2.1. Modeling Procedure and Finn Constitutive Model

In this research, the nonlinear dynamic analysis according to the effective stress to assess the pore-pressure development through earthquake loadings has been applied. The analyses have been investigated in the plane strain condition employing the FLAC finite difference package in both static and dynamic phases. For the sake of simplicity, the model has been built one step in static condition.

After determining the equilibrium condition in the static state, the dynamic phase has been utilized in the model to alter the state boundary conditions to the dynamic one and consider the earthquake loading to the system.

Regarding FLAC guidance manual, several constitutive models that facilitate soil behaviours under static and dynamic loadings have been introduced. Calculation of excess pore water pressure in the soil mass because of dynamic loading is the major factor in liquefaction phenomenon modelling process.

FLAC has a constitutive model known as the Finn model that unifies equations represented by Martin et al. [8] and Byrne [7] into the standard Mohr-Coulomb plasticity model. Through applying this model, calculation of the pore water pressure generation by calculating irrecoverable volumetric strains during dynamic analysis is possible. The void ratio in this model is considered to be constant; also it can be determined as a function of the volumetric strain and other parameters can be explained by void ratio.

Martin et al. [8] explained initially the impact of cyclic loading on raising of pore water pressure as a result of irrecoverable volume contraction in the soil mass. In these situations, since the matrix of grains and voids is filled with water, pressure of the pore water rises. Later, Byrne [7] indicated a simpler equation which corresponds to irrecoverable volume alteration and engineering shear strain with two constants. In this model, a soil mass with liquefaction potential was modelled to employ (N1)_60_ parameter as a major factor of the Finn model, so all of the soil properties of the model are required to define the program by (N1)_60_.

The reflection of waves from boundaries of the model in numerical modeling of dynamic loadings influences the results. In modeling of the quay wall, 100 meters of soil was considered on each side of the gravity quay wall so as to decrease the reflection of the waves from the lateral boundaries. From the mentioned measure, 60 meters is liquefiable sand and the rest (40 meters) is dense soil. Providing an overall stability for the model in the boundaries, the 40-meter dense layer in the boundaries of the model is considered. Consideration of the 100 meters of soil on the sides of the wall will completely attenuate the waves that propagate toward the boundaries. Even if free-field boundaries are not used, the reflection of the waves inside the model is negligible. Despite the considerable length of the model and adequate attenuation of the waves in order to make sure that the waves are not reflected inside the model, the free field is selected as the lateral boundaries.

The height of the numerical model of the gravity quay wall was decided to be 30 meters. This height was chosen both to be conventional and to have the least possible influence on the responses of the wall. In case the height of the model and accordingly the liquefiable sand layer under the wall are significantly increased, the displacement and the response of the wall will also change.

However, the height of the gravity quay wall according to the conventional sizes and measures in practice was decided to be 12 meters. It is obvious that any increase or decrease in the height of the wall influences the displacement amount as well as the liquefaction of the layers under the wall. Therefore, the height of the wall has been chosen in a way to be correspondent with the standard and conventional quantities in action.

By choosing the heights of 12 and 30 meters, respectively, for the gravity quay wall and the model, finally, there was 18 meters of soil layer under the wall that 4 meters out of which was modeled to be unliquefiable dense layer. The reason for modeling the 4-meter bottom layer, like the one mentioned for the lateral dense layers, is to provide the overall stability of the model in the boundaries. Therefore, the liquefiable sand layer has been modeled beneath the 14-meter wall. The width of the wall in the models fluctuates between 5 and 9 meters in order to study its influences. In the models designed for the study of the influences of soil improvement and its optimum range, the width is 8 meters.

**Figure 1:**
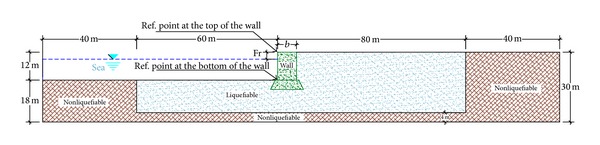
Overall geometry of the gravity quay wall (dimensions in meter, NTS), Fr and* b* = Var.

The height of water against the wall in all models is decided to be 10 meters. In Figure 1, the overall geometry of the model is displayed. Also, the reference points for the representation of the deformations of the wall are marked.

In general, two types of materials have been utilized in making the model of the quay wall: (1) concrete, (2) sand. Concrete materials have been used to make the body of the quay wall while the soil layers around the models are made of sand materials. Features of the concrete materials in all models are identical. However, features of the liquefiable sand materials in the models in which the influence of the standard penetration number is investigated are variable. Features of unliquefiable dense sand materials are identical in all models.

The analysis of the quay wall was performed in two stages. At the first stage, the model was analyzed until it reached the static equilibrium; at this stage, no external loading was considered in the model and the wall reached the equilibrium which was analyzed only under the load of seawater and embankment. At the second stage, when the model reached the static equilibrium, the seismic load was applied to the lower boundaries of the model. The seismic load was of sinusoidal wave type which was applied in the form of shear stress. The applied shear stress for the seismic load was calculated based on the selective acceleration. If the peak ground acceleration is *a*_max⁡_, the maximum shear stress (*τ*_max⁡_)_*r*_ in the depth of *h* from the surface is worked out from the following equation:

(3) $$\begin{array}{c} {\left(\tau_{\max}\right)}_{r}=\frac{\gamma h}{g}a_{\max}\cdot r_{d}. \end{array}$$

**Figure 2:**
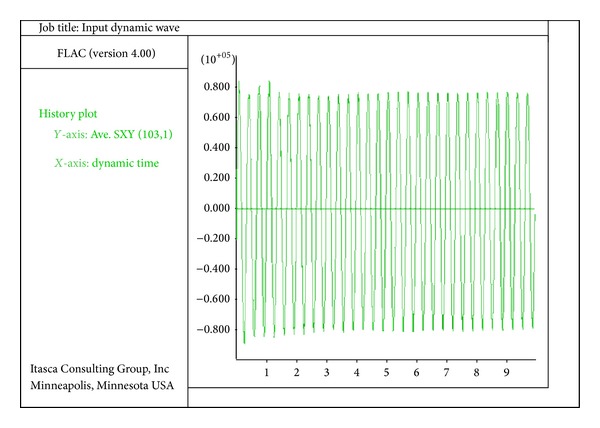
A typical seismic load applied on the base of the model (seismic acceleration is 0.2 g).

The constant *r*_*d*_ is the stress-reduction factor and is obtained through the relevant curves. The maximum seismic acceleration is 0.2 g and the frequency of the dynamic load in all models is 3 hertz. The seismic load applied in the base of the model is shown in Figure 2.

After establishment of the static equilibrium of the model and application of the proper boundary conditions and the definition of the static load, the model is analyzed at the dynamic phase. The dynamic analysis of the model has been done in undrained condition and the soil has been prevented from the drainage by the use of the capability of the software. In practice, the drainage cannot take place due to the very small duration of the dynamic loading.

As shown in Table 1, Finn model is a combined model used for the loose sand materials which are liquefied in the process of seismic loading. A brief explanation about this model is provided in the following.

During the cyclic loading, the volume of the soil decreases and such a decrease leads to an increase in the pore water pressure. The pore water pressure increases to the degree that the ratio of the pore water pressure to the total stress of the soil becomes one. In this condition, in which the effective stress has become zero, the soil undergoes liquefaction and flows. A plenty of behavioral models have so far been developed to simulate and model this state of the soil. Among the stress-strain behavioral models presented in this connection are Byrne (Booker) et al. (1976) and Martin et al. [8]. According to the research results, it has been revealed that the change in the cyclic loading is dependent on the shearing-cyclic strain amplitude and not on the shearing-cyclic stress amplitude. In the Finn combined model, (4), which has been derived according to a set of curves obtained from experiments, has been suggested by Martin and Finn et al. as follows:

(4) $$\begin{array}{c} \Delta \varepsilon_{vd}=C_{1}\left(\gamma -C_{2}\varepsilon_{vd}\right)+\frac{C_{3}\varepsilon_{vd}^{2}}{\gamma +C_{4}\varepsilon_{vd}}, \end{array}$$

in which *ε*_*vd*_ is the volume reduction under cyclic loading, is the increment in volume reduction, and *γ* is the cyclic strain amplitude.

*C*_1_, *C*_2_, *C*_3_, and *C*_4_ in the above equation are constants whose values were determined in two or three cyclic tests with fixed strain amplitude and the behavior of the volume change was completely determined under the cyclic loading. Byrne [7] has also provided a simpler equation:

(5) $$\begin{array}{c} \frac{\Delta \varepsilon_{vd}}{\gamma }=C_{1}\exp\left(-C_{2}\left(\frac{\varepsilon_{vd}}{\gamma }\right)\right), \end{array}$$

where *C*_1_ and *C*_2_ are calculated as follows:

(6) $$\begin{array}{c} C_{1}=7600{\left(D_{r}\right)}^{-2.5}. \end{array}$$

For calculation of *D*_*r*_ from the standard penetration number, the following empirical equations can be used:

(7) $$\begin{array}{c} D_{r}=15{\left(N_{1}\right)}_{60}^{0.5}. \end{array}$$

*D*_*r*_ is a relative density; therefore, the parameter *C*_1_ is as follows:

(8) $$\begin{array}{c} C_{1}=8.7{\left(N_{1}\right)}_{60}^{-1.25}. \end{array}$$

And, for the parameter *C*_2_,

(9) $$\begin{array}{c} C_{2}=\frac{0.4}{C_{1}}. \end{array}$$

To model the pore water pressure increase under undrained cyclic loading in the FLAC software, both (4) and (5) have been added to the Mohr-Coulomb plastic model in accordance with the above explanations in order to make a new model named Finn.

### 2.2. Boundary Condition

**Figure 3:**
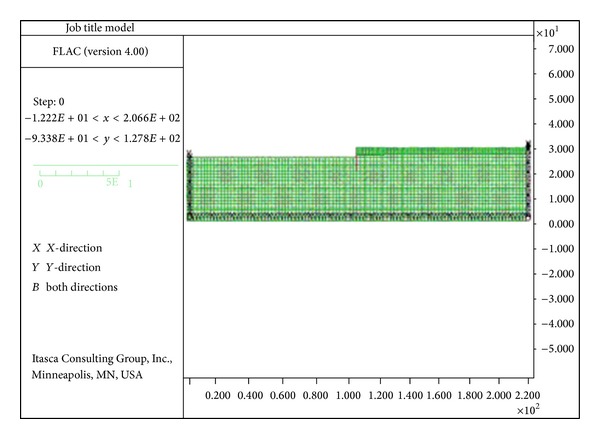
The selected finite difference mesh for numerical analysis by FLAC 2D.

Figure 1 presents the geometry and general dimension of the developed model in FLAC to do parametric research. The finite mesh chose to investigate the numerical analyses as shown in Figure 3. The base boundary of the model embedded along horizontal and vertical directions in both static and dynamic analyses. Regarding statistical analysis, right and left boundaries of the mesh were horizontally fixed. In dynamic analyses, enough distance between the structure and right and left boundaries should be determined to prohibit the reflection of waves contacting the boundaries. Selection of sufficient dimensions for the model plays a significant role in modeling process (Figure 3).

## 3. Results and Discussion

**Figure 4:**
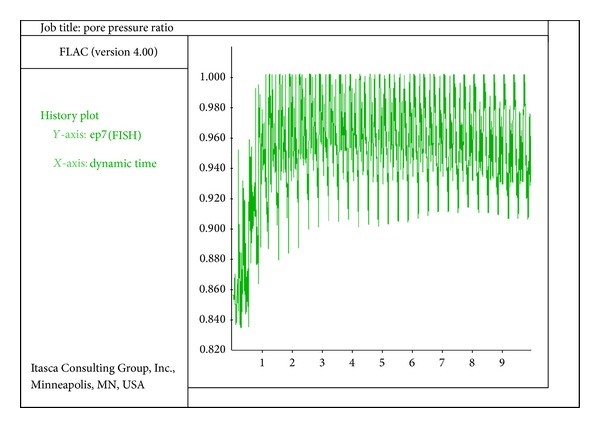
Ratio of pore water pressure versus time for the element on the front of the wall under the sea floor (time: seconds).

**Figure 5:**
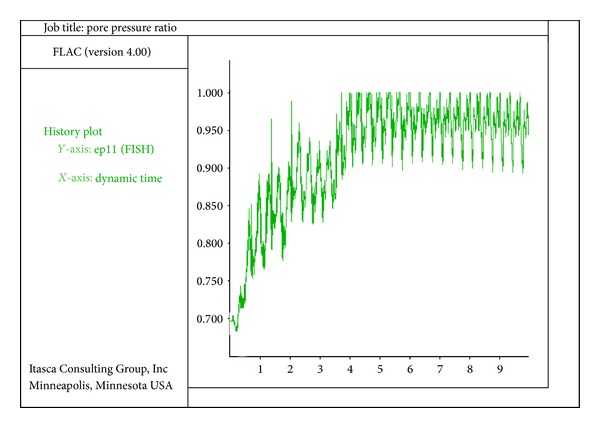
Ratio of pore water pressure versus time for the element on the back of the quay wall (time: seconds).

The deformation of the quay walls of the model during the process of liquefaction of the backfill and bottom soil is considerable. Deformation of the walls of the model is of horizontal, vertical, and rotational displacements. In Figure 4, the ratio of pore water pressure in the front side of the wall under the seafloor is shown. In Figure 5, the diagram shows the ratio of pore water pressure versus the time for the element in the back side of the quay wall. The study of the liquefaction of the soil around the quay wall of the analytical models shows that no liquefaction takes place in the areas exactly located under the wall due to the weight of the wall and, in consequence, the enormousness of the stress. In the areas behind the wall that are at the levels of the bottom of the wall, the ratio of pore water pressure or liquefaction potential proportionally increases as it gets farther from the quay wall. Of course, this status is not seen in all models; however, the review of the models implies that the farther getting away from the quay wall, the greater excess pore water pressure. In comparison, the liquefaction in the areas behind the wall starts later than that of the areas in front of the wall (under the seafloor).

### 3.1. Effect of the Width of the Wall

**Figure 6:**
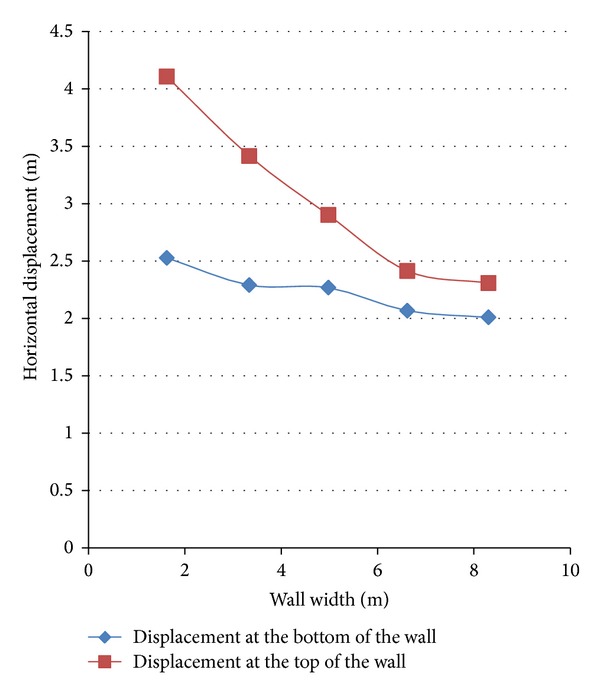
Diagram of the changes in horizontal displacement of the wall versus the width of the wall.

In order to study the effect of the width of the wall on decreasing the dynamic displacements of the wall, its width was changed between 5 and 9 meters. At the first stage, the static analysis was performed and, after the establishment of the static equilibrium, the values for the displacements of the wall in the memory of the software were manually changed to zero and the dynamic loading was applied. Based on the above explanation, the effect of the width of the wall on the dynamic displacements can thoroughly be studied. In Figure 6, the diagram of the changes on horizontal displacement of the wall at the top and bottom points against the wall width is shown. It is clearly understood that the increase in the width of the wall cannot control the horizontal displacement of the wall and the rate of displacement decrease of the wall reduces from the widths above 7 meters. In general, the dynamic displacement of the gravity wall is the result of two factors. The first factor is the increase in the lateral pressure from the embankment which is the result of the seismic loading and the second one is the decrease in the stability of the wall as a result of soil liquefaction around the wall. The stability of the wall against the lateral pressures may be the reason for the significant decrease in the horizontal displacement of the wall with the increase of the width of the wall in the first parts of the above diagram. But the part of the displacement of the wall which is the result of the liquefaction areas around the wall cannot be noticeably controlled with the increase of the width of the wall, or perhaps the cause of no decrease in the horizontal displacement of the wall above the heights of 7 meters is the above-mentioned reason. Also, the diagram shows that the horizontal displacement of the lower part of the wall does not change dramatically with the increase in the width of the wall which ensures the authenticity of the aforesaid notion. A comparison of the horizontal displacement curves reveals that in high latitudes (approximately 8 to 9 meters) the displacement of the wall is an integrated transfer and the rate of the rotation of the wall decreases.

According to Diagram 7, the vertical displacement of the wall with the increase in the width of the wall makes no significant change. The diagram also shows the vertical settlement of the embankment areas near the wall. As it is shown in the diagram, the settlement of the embankment near the wall also converges at a constant rate by increasing the width of the wall which is in accordance with the convergence of the horizontal displacement of the wall.

### 3.2. Effect of the Standard Penetration Number

Some models of the wall with the standard penetration numbers between 13 and 40 were modeled to study the effect of the standard penetration number on the stability of the gravity quay wall.  Figure 8 shows that, with the increase of the standard penetration number, the quay wall displacement noticeably decreases. The diagram also shows that the effect of the increase in SPT on the horizontal displacement is more than that of the vertical displacement.

**Figure 7:**
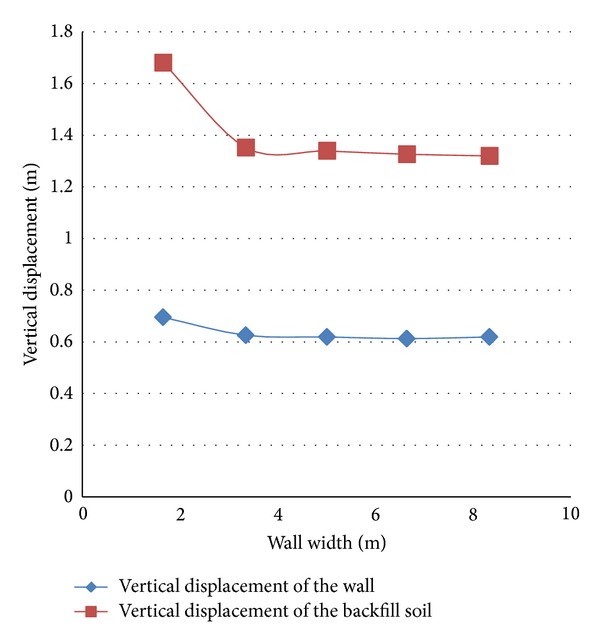
Vertical displacement of the wall and the embankment.

**Figure 8:**
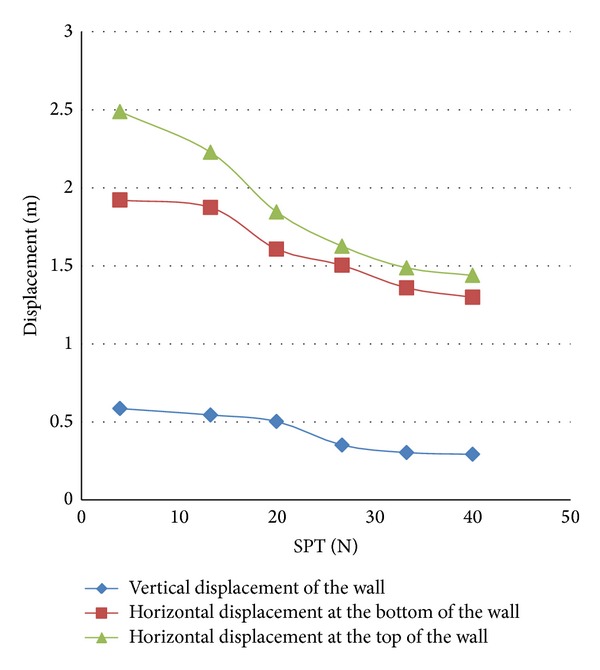
Horizontal and vertical displacements of the quay wall versus the SPT number for the wall of 8 meters wide.

As can be seen in Figure 8, the effect of increasing the standard penetration number of the soil is noticeable when the number exceeds 20; for example, the transition from 20 to 25 brings about a step or refraction. The diagrams also show that the effect of the standard penetration number greater than 35 is practically attenuated and the diagram of the displacement of the wall tends to be constant. The comparison of the diagrams of the horizontal displacement of the upper and lower parts of the quay wall supports the idea that the increase of the standard penetration number not only decreases the horizontal displacement but also minimizes dramatically the rotational displacement of the wall. Based on Figure 7 on SPT number of 35, the horizontal displacement of the upper and lower parts of the quay wall becomes almost equal which indicates that no rotation has taken place and the wall has undergone a complete horizontal displacement.

### 3.3. Optimum Area for Soil Improvement

Since improvement of soil incurs huge costs and there are plenty of difficulties in implementation of such amendment particularly in littoral areas, knowing the optimum and adequate range for the soil improvement is immensely important. Therefore, the optimum range for the soil improvement is studied in three areas. The areas in question are as follows:

- the soil improvement area* behind* the quay wall;
- the soil improvement area* under* the quay wall;
- the soil improvement area* in front of* the quay wall.

**Figure 9:**
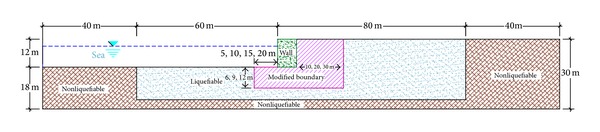
Areas selected to determine the optimum range for the soil amendment.

The soil improvement has the standard penetration number of 35. The soil behind, under, and in front of the quay wall has been improved, respectively, at the paces of 10, 20, and 30 meters from the quay wall, at the paces of 6, 9, and 12 meters deep, and at the paces of 5, 10, 15, and 20 meters from the quay wall.  Figure 9 illustrates the soil improvement areas.

It should be mentioned that the depth of the improved layer of the soil in front of the quay wall has been decided to be 9 meters while the width of the soil improved area under the quay wall has been decided to be 20 meters and the depth of the improved area like the height of the wall has been decided to be 12 meters. When the effect of the soil improvement in front of the wall was studied, the improvement was not done on the other fronts so that their effects could not interfere with the soil properties of the front under the study and the soil properties in the other two areas were decided to be like those of the liquefied soil.

**Figure 10:**
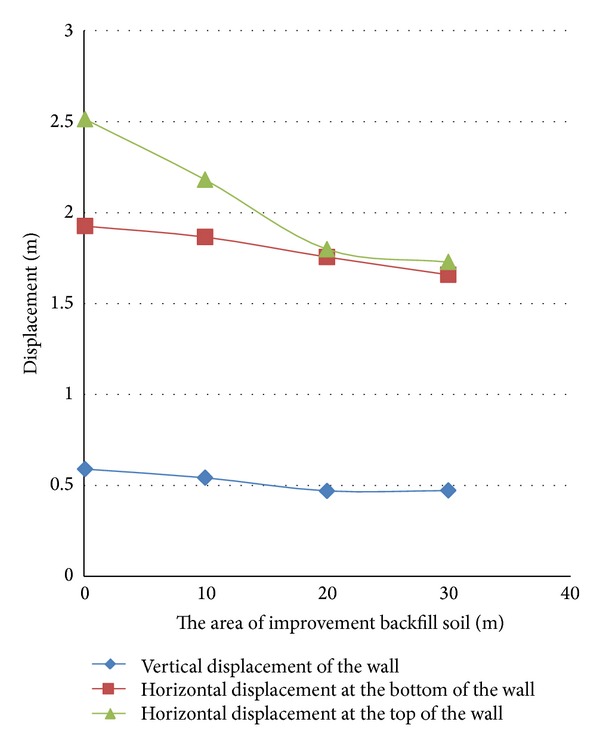
Horizontal and vertical displacements of the quay wall in different soil improvement areas behind the wall.

**Figure 11:**
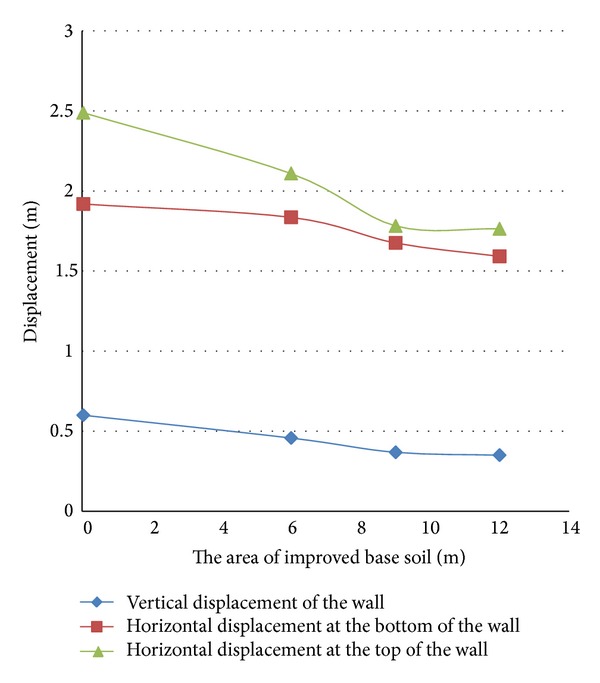
Horizontal and vertical displacements of the quay wall in different soil improvement areas under the wall.

**Figure 12:**
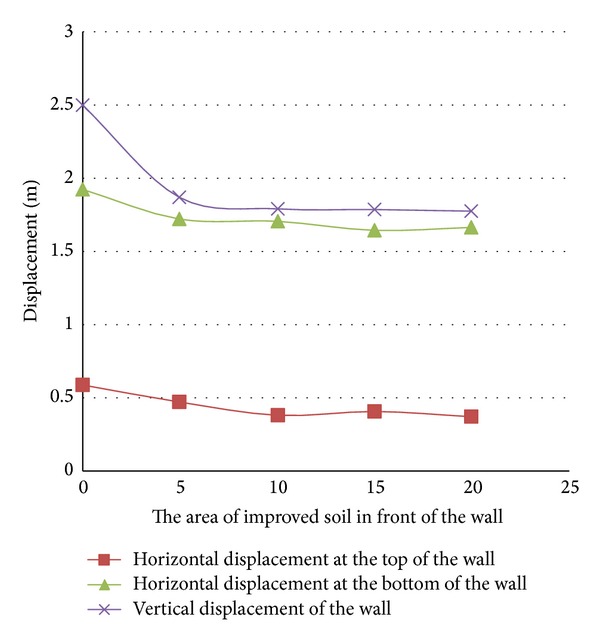
Horizontal and vertical displacements of the quay wall in different soil improvement areas in front of the wall.

The results have been shown in Figures 10 to 12.

Zero in the horizontal axes of the above figures reveals the results of no improvement in the soil.

The results of the horizontal displacement of the upper and lower points of the wall and its vertical settlement show the optimum and adequate range of the soil improvement in each area under the study.

Studying the diagrams reveals the following results.

- As can be seen in Figure 10, when the improvement of the soil behind the wall is the focus of the study, for a wall with the aforesaid dimensions, soil improvement for a length of 20 meters from the wall has the maximum adequacy in decreasing the displacements of the wall and any further soil improvement beyond the mentioned length will not have a significant effect in this regard. The diagrams also reveal that the horizontal displacement of the top of the wall has been affected more by the improvement of the embankment behind the wall than the horizontal displacement of the lower part or the vertical settlement.
- Studying Figure 11 reveals that when the improvement of the soil under the wall is the focus of the study, soil improvement in depths over 9 meters has no effect on the control of the wall displacements. This figure also shows that the effect of the soil improvement of the bottom has considerable effect on the horizontal displacement of the upper part of the wall. Of course, based on the diagram, the vertical settlement of the wall for the state of the soil improvement up to a depth of 9 meters is half of that in the state of unimprovement.
- If the three diagrams of Figure 12 are studied simultaneously, it is understood that improvement of the soil in front of the wall up to a length of 8 meters decreases the wall displacements. Any further soil amendment beyond the said length does not have a noticeable effect on the deformations; furthermore, there are plenty of problems in such further improvement.

## 4. Conclusions

The conclusions of the paper are summarized as follows.

1. Increase in the width of the wall up to certain measures will affect the decrease in the deformations of the wall. Increase in the width of the wall more than the said measures will have no effect on decreasing the displacements. The studies on a wall of 12-meter height show that the horizontal and vertical displacements of the wall have not significantly decreased with increasing the width of the wall over 7 meters. The only continuous effect of the increase in the width of the wall is the decrease in the rotation of the wall; that is, the difference between the displacements of the upper and the lower points decreases with increasing the width of the wall. In heights of approximately more than 9 meters, the horizontal displacement of the wall is in fact a full transfer with very little rotation.
2. Increasing the standard penetration number of the soil around the quay wall decreases the displacements of the quay wall. The effect of the increase of the standard penetration number of the soil on the wall displacement is noticeable when the number goes over 20. Also, the effect of the standard penetration number in the numbers above 35 is practically attenuated and the diagram of the displacement of the wall tends to be constant.
3. As for the optimum range of the soil improvement for the gravity quay wall which was studied, it should be mentioned that the soil improvement behind the wall up to a length of 20 meters from the wall decreases the displacements of the wall to a great extent. Improvement of the soil behind the wall for more than the said length has no effect on decreasing the wall displacements. Improvement of the soil under the wall for a depth of approximately 9 meters decreases the wall displacements. Soil improvement in depths over 9 meters has no noticeable effect on the control of the wall displacements. As for the improvement of the soil in front of the wall, the optimum range for improvement is approximately 8 meters from the wall. Generally, the effect of the soil improvement in the bottom and front parts of the wall on decreasing the vertical settlement of the wall is more than the effect that happens in the soil improvement behind the wall.

## Figures and Tables

**Table 1:** Features of the materials used in the analytical models of the gravity quay wall.

Materials	Behavior model	Dry density (Dry unit weight) (Kg/m^3^)	Relative density (*D*_*r*_ %)	Void ratio	Porosity	Angle of internal friction	SPT	Shear modulus (MPa)	Bulk module (MPa)
1	Finn	1600	47	1	0.500	32	10	6	10

2	Finn	1530	55	0.704	0.413	36.5	13	6	10
	Finn	1559	67	0.673	0.402	38.5	20	8	13.33
	Finn	1575	75	0.650	0.394	40	25	9.2	15.33
	Finn	1586	82	0.630	0.387	41.5	30	10	16.66
	Finn	1591	89	0.610	0.79	42.9	35	11.2	18.66
	Finn	1595	94.86	0.597	0.374	43.4	40	12	20

3	Mohr-Coulomb	2000	—	—	—	42	—	12	20

4	Elastic	2400	—	—	—	—	—	8300	11100

## References

[1] JGS: liquefaction-induced flow of ground and its effect to structures.

[2] Hamada M, Yasuda S, Isoyama R, Emoto K (1986). Observation of permanent displacements induced by soil liquefaction. *Journal of Geotechnical Engineering*.

[3] Mirhosseini SM (1993). *Soil Dynamics*.

[4] Richards R, Elms DG (1979). Seismic behavior of gravity retaining walls. *ASCE Journal of the Geotechnical Engineering Division*.

[5] Madabhushi S, Zeng X (1998). Seismic response or gravity quay walls. II: numerical modeling. *Journal of Geotechnical and Geoenvironmental Engineering*.

[6] Cundall PA, Roth W, Scott R Fast lagrangian analysis of continua manual.

[7] Byrne P A cyclic shear-volume coupling and pore-pressure model for sand.

[8] Martin GR, Finn WDL, Seed HB (1975). Fundamentals of liquefaction under cyclic loading. *Journal of Geotechnic Division ASCE*.

[9] Liou CP, Richart FE, Streeter VL (1977). Numerical model for liquefaction. *Journal of the Geotechnical Engineering Division, ASCE*.

[10] Finn WDL, Lee KW, Martin GR (1977). An effective stress model for liquefaction. *ASCE Journal of the Geotechnical Engineering Division*.

[11] Katsikas CA, Wylie EB (1982). Sand liquefaction: inelastic effective stress model.. *Journal of the Geotechnical Engineering Division, ASCE*.

[12] Desai CS (2000). Evaluation of liquefaction using disturbed state and energy approaches. *Journal of Geotechnical and Geoenvironmental Engineering*.

[13] Liyanapathirana DS, Poulos HG (2002). A numerical model for dynamic soil liquefaction analysis. *Soil Dynamics and Earthquake Engineering*.

[14] Lee KL, Seed HB (1967). Cyclic stress conditions causing liquefaction of sand. * Journal of the Soil Mechanics and Foundations Engineering Division*.

[15] Elgamal A, Zeghal M, Parra E (1996). Liquefaction of reclaimed island in Kobe, Japan. *Journal of Geotechnical Engineering*.

[16] Ashford SA, Rollins KM, Lane D (2004). Blast-induced liquefaction for full scale foundation testing. *Journal of Geotechnical and Geoenvironmental Engineering, ASCE*.

[17] Seed HB, Idriss IM (1982). *SoilLiquefaction during Earthquakes*.

[18] Tokimatsu K, Seed HB (1987). Evaluation of settlement in sand due to earthquake shaking. *Journal of the Geotechnical Engineering Division*.

[19] Robertson PK, Wride CE (1998). Evaluating cyclic liquefaction potential using the cone penetration test. *Canadian Geotechnical Journal*.

[20] Youd TL, Idriss IM (2001). Liquefaction resistance of soils: summary report from the 1996 NCEER and 1998 NCEER/NSF workshops on evaluation of liquefaction resistance of soils. *Journal of Geotechnical and Geoenvironmental Engineering*.

[21] Idriss IM, Boulanger RW Semi-empirical procedures for evaluating liquefaction potential during earthquakes.

[22] Rollins KM, Seed HB (1990). Influence of buildings on potential liquefaction damage. *Journal of Soil Mechanics and Foundations Engineering Division*.

[23] Peacock WH, Seed HB (1968). Sand liquefaction under cyclic loading simple shear conditions. *Journal of the Soil Mechanics and Foundations Division*.

[24] Mulilis JP, Chan CK, Seed HB (1975). The effects of method of sample preparation on the cyclic stress strain behavior of sands. *Report*.

[25] Hynes ME, Olsen R Influence of confining stress on liquefaction resistance.

[26] Seed HB, Idriss IM (1971). Simplified procedure for evaluating soil liquefaction potential. *Journal of Geotechnic Division*.

[27] Ishihara K, Takatsu H (1979). Effects of over-consolidation and K_0_ conditions on the liquefaction characteristics of sands. *Soils and Foundations*.

